# Managing the Skin Microbiome as a New Bacteriotherapy for Inflammatory Atopic Dermatitis

**DOI:** 10.7759/cureus.48803

**Published:** 2023-11-14

**Authors:** Dian Andriani Ratna Dewi, Angki Perdiyana, Ni M Wiliantari, Farrasila Nadhira, Nabila Arkania, Cut A Salsabila, Clara V Allun, Arohid Allatib, Kelvin Dewantara

**Affiliations:** 1 Department of Dermatovenereology, Faculty of Military Medicine, The Republic of Indonesia Defense University, Bogor, IDN; 2 Department of Dermatovenereology, Gatot Soebroto Central Army Hospital, Central Jakarta, IDN; 3 Department of Dermatovenereology, Ratna Dewi Principal Clinic, Bekasi, IDN; 4 Department of Dermatovenereology, Faculty of Medicine, Public Health, and Nursing, Gadjah Mada University, Special Region of Yogyakarta, IDN

**Keywords:** microbial dysbiosis, therapeutic approve, skin and gut microbiome, immune system and inflammation, atopic dermatitis, skin barrier

## Abstract

The microbiome, comprising various bacteria, assumes a significant role in the immune system's maturation and maintaining bodily homeostasis. Alterations in the microbial composition can contribute to the initiation and progression of inflammation. Recent studies reveal that changes in microbial composition and function, known as dysbiosis in the skin and gut, have been associated with altered immunological responses and skin barrier disruption. These changes are implicated in the development of several skin diseases, such as atopic dermatitis (AD). This review examines research demonstrating the potential of microbiome repair as a therapeutic approach to reduce the effect of inflammatory processes in the skin during atopic dermatitis. This way, corticosteroids in atopic dermatitis therapy can be reduced or even replaced with treatments focusing on controlling the skin microbiome. This study used scientific literature from recognized platforms, including PubMed, Scopus, Google Scholar, and ScienceDirect, covering publications from 2013 to 2023. The primary aim of this study was to assess the efficacy of skin microbiome management in treating atopic dermatitis. This study concludes that physicians must comprehensively understand the microbiome's involvement in atopic dermatitis, including its pathophysiological implications and its relevance to therapeutic interventions.

## Introduction and background

Atopic dermatitis (AD) is a chronic inflammatory condition characterized by recurring skin lesions and itchiness. In the majority of cases, the primary symptoms of AD become apparent during the first five years of an individual's existence. Around 25% of these children persist in experiencing AD throughout their adult years [1]. By the time children reach preschool age, approximately 30% of those diagnosed with AD exhibit allergic reactions to certain types of food, such as eggs, cow's milk, and peanuts. Individuals exhibiting moderate to severe AD are associated with a 50% probability of acquiring asthma and a 75% probability of developing hay fever [2].

The etiopathogenesis of AD is a multifaceted process that involves a combination of hereditary and environmental factors. These factors contribute to the development of aberrant immune system responses, specifically an increased prevalence of CD4 cell differentiation towards the Th2 lineage. The outcome of heightened activity of Th2 lymphocytes is an amplified secretion of cytokines, particularly IL-4, IL-5, and IL-13, while simultaneously reducing the production of IFN-gamma. Individuals afflicted with AD commonly exhibit an immune response characterized by IgE-mediated hypersensitivity to extrinsic and intrinsic antigens. It is widely believed that environmental variables, including exposure to indoor and outdoor allergens and pollution during the perinatal period, diet, and the microbiome, play a significant role in influencing the development and intensity of AD [3].

Research has revealed that various microorganisms, encompassing bacteria, yeast, and viruses, cohabitate within different human body regions, such as the intestines, skin, lungs, and oral cavity. The term "microbiota" can be traced back to the early 1900s [4]. Moreover, the human microbiota, also known as “the hidden organ,” contributes more than 150 times more genetic information than the entire human genome [5]. The terms "microbiota" and "microbiome" are sometimes employed synonymously, yet distinct dissimilarities exist between these concepts. Microbiota refers to the collection of live microorganisms that inhabit a specific area, such as the microbiota in the oral cavity and gastrointestinal tract. The term "microbiome" encompasses the genetic material microbes possess within a given environment. This includes the community of microorganisms and other aspects of microbial structure, metabolites, and environmental factors. The microbiome comprises a broader range of organisms than the microbiota [6].

**Figure 1:**
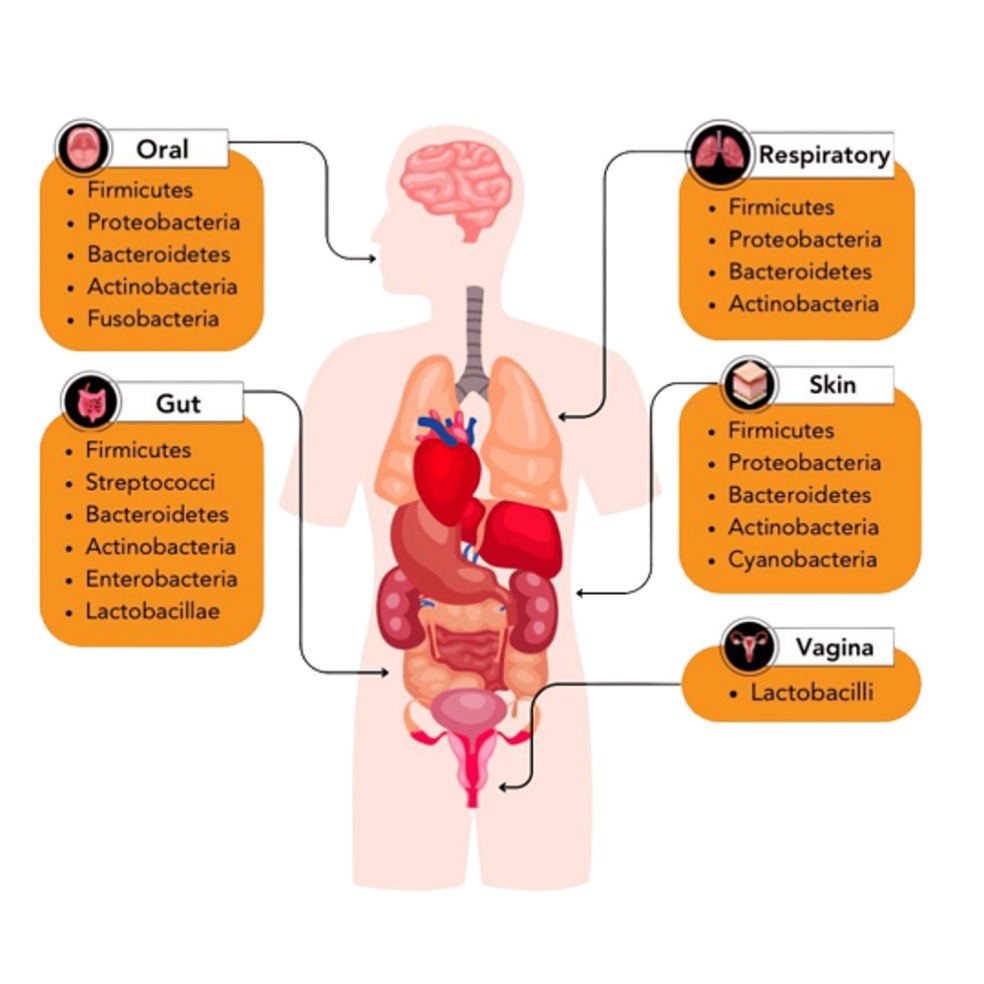
The microbial makeup exhibits variation across different anatomical sites of the human body. This figure was created by AP, one of the authors of this article with Biorender.com.

The microbial mix exhibits variability across different anatomical sites (Figure 1). The gut microbiota comprises six phyla: Firmicutes, Bacteroidetes, Actinobacteria, Proteobacteria, Fusobacteria, and Verrucomicrobia. Firmicutes and Bacteroidetes are the predominant species among these [7]. The oral microbiota is widely recognized as the second most enormous microbial population within the human body. The oral cavity can be subdivided into many habitats of microbiota, encompassing saliva, tongue, tooth surfaces, gums, buccal mucosa, palate, and subgingival/supragingival plaque. Typically, the predominant bacterial taxa within the oral microbiota encompass Firmicutes, Proteobacteria, Bacteroidetes, Actinobacteria, and Fusobacteria [8]. The location and diversity of glands and hair follicles in human skin exhibit variations across different geographic regions. The unique microbial communities in other skin regions can be attributed to the inherent physical and chemical variations between these locations. The skin microbiota typically consists of Actinobacteria, Bacteroidetes, Cyanobacteria, Firmicutes, and Proteobacteria [9].

Extensive research has been conducted on the skin microbiome, which has been established to impact innate immunity significantly [5]. Interactions between cellular immunity and the microbiome have been observed in several tissues, such as the gastrointestinal tract [10]. The abundance and composition of microorganisms should exhibit a proportional level of diversity, encompassing both numerical and taxonomic aspects, to maintain a state of equilibrium within the microbiota ecosystem, sometimes referred to as eubiosis. Dysbiosis is an ecological state when the bacterial population no longer coexists in mutually advantageous symbiosis [11].

Preserving an undamaged dermal barrier and promoting various skin microbiota are crucial for optimal skin health. Alterations in the microbial composition of the skin can contribute to the initiation and progression of inflammation. Certain skin illnesses, such as AD, can be attributed to alterations in the variety of the skin microbiome. These changes are characterized by an elevated presence of pathogenic microbiomes, leading to a persistent inflammatory response [12]. Numerous elements can influence the manifestation of AD, encompassing impairment of the epidermal barrier, innate and adaptive immune responses, and the composition of the skin microbiota. Numerous studies have established a correlation between the skin microbiota and many parameters, including age, lesions, the bacterial composition of AD lesions, the concentration of sebaceous glands, humidity, temperature, hereditary factors, and environmental influences [13]. The commensal microbiome plays a crucial role in preserving the integrity of the skin barrier by facilitating vital physiological activities within the skin. This paper aims to explore therapeutic approaches focused on inducing eubiosis of the skin microbiota, considering its impact on the development of AD [5].

Search strategies

**Figure 2:**
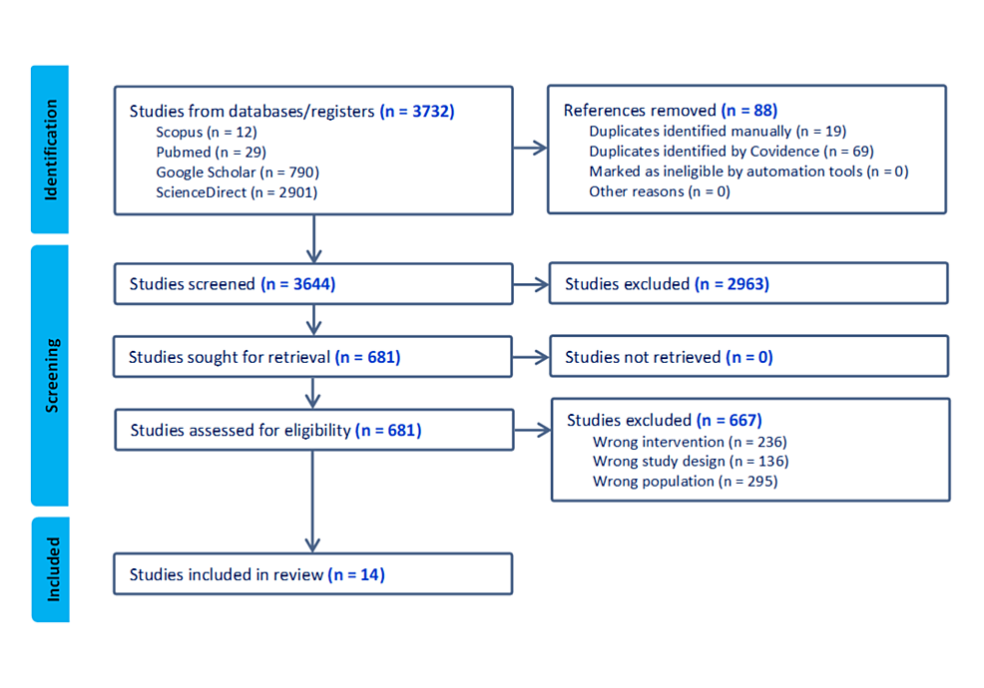
Preferred reporting items for systematic reviews and meta-analysis (PRISMA) flow diagram. This figure was created by FN, one of the authors of this article.

The methodology employed in this systematic review followed the recommendations outlined in the Preferred Reporting Items for Systematic Reviews and Meta-Analysis (PRISMA) statement, which offers a standardized structure for reporting systematic reviews. A thorough examination of the existing literature was conducted by searching databases such as PubMed, Google Scholar, Scopus, and ScienceDirect. The search query employed essential terms, namely microbiome, AD, skin barrier, and randomized controlled trials (RCTs), to identify pertinent research studies. The preliminary investigation was carried out on August 10, 2023, with no additional or correlated findings (Figure 2).

This study's inclusion criteria are the research focusing on managing the microbiome as a therapy approach for AD, the study with a randomized controlled trial study design, and a peer-reviewed article published between 2013 and 2023. This study's exclusion criteria are a non-randomized controlled trial, observational articles, and articles that only explain AD.

Data extraction

The titles and abstracts were comprehensively scrutinized to verify their conformity with the pre-established inclusion criteria. The comprehensive reports were further assessed to determine if the articles satisfied the inclusion criteria, which comprised several factors such as outcomes, interventions, research designs, and patient demographics. The justifications for the exclusion of research were clarified.

Quality assessment

We evaluated the 14 studies that served as the basis for the quality of our review. Two independent reviewers (CVA and CAS) assessed the methodological quality of the research using the Risk of Bias 2 (RoB2) methodology. Cochrane Reviews exclusively consist of randomized studies subjected to assessment of their risk of bias using the Cochrane risk-of-bias methodology for randomized trials, version 2. The established classifications of bias in the RoB 2 framework encompass a diverse range of potential concerns about clinical trials and their dissemination. A set of inquiries called "signaling questions" aims to gather data on trial features pertinent to the risk of bias in each domain. The program presents a risk assessment of preference for each part by analyzing the responses to the signaling questions. The ratings for bias risk can range from "low" to "high," with the inclusion of an intermediate option labeled "some concerns." From 14 RCT studies, we discovered 12 studies with low risk and two studies with moderate risk. So, for the most part, we used high-caliber or low-bias publications in this study. Because of the common findings in the area that evaluate bias from sources, confounders, participant selection, and departures from the intended intervention, these publications are of high quality and are categorized as low-bias studies.

Description of selected studies

**Figure 3:**
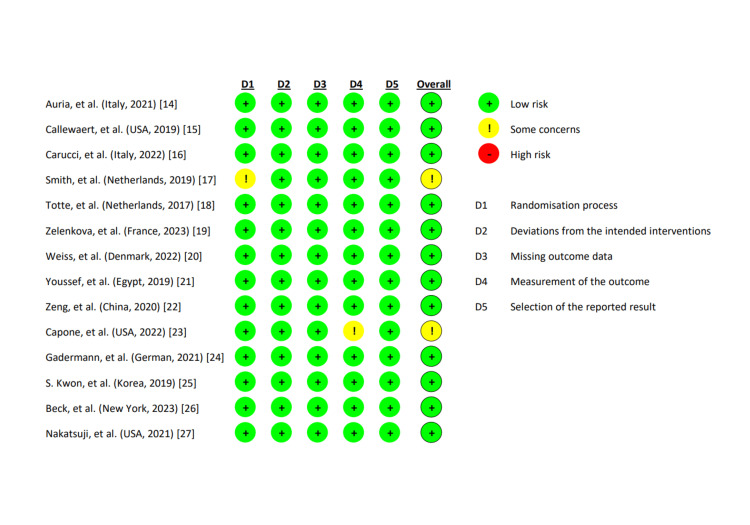
Quality assessment of selected studies using cochrane risk-of-bias technique for randomized trials, version 2. Summary of study quality assessment of the randomized controlled trial [14-27]. This figure was created by the CVA of the authors of this article.

The results of the systematic search yielded a total of 28 findings. This study exclusively included scholarly papers written in English and subjected to peer review, with publication dates ranging from 2013 to 2023. The datasets undergo a procedure to eliminate a singular occurrence of duplicated research. A comprehensive analysis was conducted on 32 articles, wherein their titles and abstracts were thoroughly examined. This process led to the further review of 26 full-text studies. Twelve studies were excluded from the analysis due to discrepancies in the intervention (n = 4) because they did not address atopic dermatitis therapy focused on skin microbiota and discrepancies in the research design (n = 8) because they were not RCTs. This analysis comprised a total of 14 papers. Details on further examination are mentioned in Figure 3 and Table 1.

**Table 1:** Summary of included study characteristics. SCORAD: scoring atopic dermatitis index, MRSA: methicillin-resistant S. aureus, NB-UVB: narrowband ultraviolet B, EASI: Eczema Area and Severity Index, CFU: colony-forming units, TCS: topical corticosteroid, TISS: Three-Item Severity Score, IL-13: interleukin-13, ShA9: *S. hominis* A9, ShA9 DNA: *S. hominis* A9 deoxyribonucleic acid, mRNA: messenger ribonucleic acid, PSMα: phenol-soluble modulin-alpha, CoNS: coagulase-negative staphylococcus.

No.	Author (country in which the study conducted)	Title	Aim	Population and intervention	Results and outcomes
1.	Auria et al. (Italy) [14]	Rice flour fermented with *Lactobacillus paracasei* CBA L74 in the treatment of atopic dermatitis in infants: A randomized, double-blind, placebo-controlled trial (2021)	To assess the effect of a fermented rice-flour obtained from *L. paracasei* CBA L74 in managing infants with moderate to severe atopic dermatitis	There were 25 subjects per group Infants with moderate to severe atopic dermatitis, aged 6–36 months, were randomly assigned to receive once-daily consumption of rice flour containing heat-killed probiotic *L. paracasei* CBA L74 or placebo for 12 weeks as supplementary approach to topical treatment.	The SCORAD index decreased in both groups. The use of topical steroids significantly decreased in the experimental group compared to the placebo group. In conclusion, though the heat-killed *L. paracasei* was not proven to be effective in reducing the severity of atopic dermatitis, it showed a steroid-sparing effect, the value of which needs to be further investigated.
2.	Callewaert et al. (USA) [15]	IL-4Rα Blockade by Dupilumab Decreases Staphylococcus aureus Colonization and Increases Microbial Diversity in Atopic Dermatitis (2019)	To investigate the effects of Dupilumab, a medication used to treat atopic dermatitis on the bacterial colonization of the skin, specifically focusing on *Staphylococcus aureus.*	The population in these studies consisted of 54 patients with atopic dermatitis. The intervention was the administration of Dupilumab 200 mg weekly.	Dupilumab treatment significantly reduced the abundance of *S. aureus* on the skin of atopic dermatitis patients. This decrease was observed as early as week four and was sustained until week 16.
3.	Carucci et al. (Italy) [16]	Therapeutic effects elicited by the probiotic *Lacticaseibacillus rhamnosus* GG in children with atopic dermatitis. The results of the ProPAD trial | Enhanced Reader (2022)	To discuss the therapeutic effects of the probiotic *Lacticaseibacillus rhamnosus* on atopic dermatitis, with a particular focus on the results of the ProPAD trial.	A hundred patients with atopic dermatitis. The intervention involved the administration of systemic the probiotic *Lacticaseibacillus rhamnosus *capsule containing 1 × 10^10^ CFU once a day.	The results of the ProPAD trial demonstrated a significant improvement in the symptoms of atopic dermatitis in the group treated with *Lacticaseibacillus rhamnosus* compared to the control group. The outcome suggests that the probiotic *Lacticaseibacillus rhamnosus* could be a potential therapeutic treatment for atopic dermatitis.
4.	Smits et al. (Netherlands) [17]	Targeting the Cutaneous Microbiota in Atopic Dermatitis by Coal Tar via AHR-Dependent Induction of Antimicrobial Peptides (2019)	To investigate the effect of coal tar treatment on the composition of the cutaneous microbiome in patients with atopic dermatitis.	They included ten healthy volunteers and seven patients with atopic dermatitis. The intervention was the treatment with coal tar, a traditional therapy for atopic dermatitis.	Coal tar treatment significantly reduced many phylogenetically related *S. aureus* and *S. capitis* taxa in the cutaneous microbiome of patients with atopic dermatitis. The increase in *S. epidermidis*, a health-associated commensal, was much larger for coal tar treatment than for vehicle treatment.
5.	Totte et al. (Netherlands) [18]	Targeted anti-staphylococcal therapy with endolysins in atopic dermatitis and the effect on steroid use, disease severity, and the microbiome: study protocol for a randomized controlled trial (MAAS trial) | Enhanced Reader (2017)	To evaluate the efficacy and safety of targeted anti-staphylococcal therapy with endolysins in treating atopic dermatitis caused by *S. aureus*.	A total 100 participants with atopic dermatitis, Staphefekt, in a cetomacrogol-based cream or a placebo gave to experimental population for 12 weeks, followed by an 8-week follow-up period.	The result of this trial provides data about the effect of long-term anti-staphylococcal therapy with staphefekt on corticosteroid and quality of life in patients with moderate to severe atopic dermatitis.
6.	Zelenkova et al. (France) [19]	Impact of daily use of emollient ‘plus’ on corticosteroid consumption in patients with atopic dermatitis: An open, randomized controlled study | Enhanced Reader (2023)	To investigate the impact of daily use of an emollient 'plus' on corticosteroid consumption in patients suffering from atopic dermatitis.	One hundred thirty patients suffering from dermatitis. The intervention was the daily use of an emollient 'plus'.	The daily use of the emollient 'plus' significantly reduced corticosteroid consumption in comparison to the control group. This suggests that the emollient 'plus' could be an effective intervention for managing atopic dermatitis and reducing reliance on corticosteroids.
7.	Weiss et al. (Denmark) [20]	Topical niclosamide (ATx201) reduces *S. aureus* colonization and increases Shannon diversity of the skin microbiome in atopic dermatitis patients in a randomized, double‐blind, placebo‐controlled Phase 2 trial (2022)	To investigate the effectiveness of topical niclosamide (ATx201) in reducing* S. aureus* colonization and increasing the diversity of the skin microbiome in patients with atopic dermatitis.	The study population is 40 atopic dermatitis patients consisting of male and female subjects between 18 and 70 years of age. The intervention was the application of ATx201 Ointment 2%, a topical formulation of the drug niclosamide, traditionally used to treat tapeworm infections. The ointment was applied to selected treatment areas on the patient's skin.	ATx201 was a potent agent against MRSA and other strains of *S. aureus*, including those resistant to commonly used topical treatments. It exhibited bacteriostatic killing kinetics at lower concentrations, but at a higher concentration, it cleared the bacterial population.
8.	Youssef et al. (Egypt) [21]	Glycerol 85% efficacy on atopic skin and its microbiome: a randomized controlled trial with clinical and bacteriological evaluation (2019)	To evaluate and compare the efficacy and tolerability of concentrated glycerol 85% and NB-UVB in treating atopic dermatitis.	The total population was 45 involving 30 patients with mild to moderate atopic dermatitis and 15 healthy volunteer, 18 males (40%) and 27 females (60%), with ages ranging from 6 to 37 years and a mean age of 12.1±7.3 years.	The results of the study showed that both glycerol and NB-UVB achieved a significant reduction of clinical scores; SCORAD and itch score; after four weeks in comparison to baseline. The clinical effectiveness, as defined by achievement of SCORAD50 and ITCH50 at the end of treatment, was comparable between both groups.
9.	Zeng et al. (China) [22]	Topical ozone therapy restores microbiome diversity in atopic dermatitis (2020)	To investigate the effects of topical ozone therapy on the severity of atopic dermatitis and the diversity of the skin microbiome in atopic dermatitis lesions.	The studies involved 12 patients with atopic dermatitis, consisting of 4 males and eight females ranging from 6 to 28 years old. The intervention was the application of topical ozone therapy to the atopic dermatitis lesions.	The results of the studies showed that topical ozone therapy significantly reduced the severity of atopic dermatitis in patients. The SCORAD dropped by 22.15% after three days of treatment. The modified EASI decreased by 48.0 ± 18.6% in the treatment group.
10.	Capone et al. (USA) [23]	A randomized clinical study on the effects of emollient use on the developing infant skin microbiome and metabolome (2022)	To investigate the effects of emollient use on the developing infant skin microbiome and metabolome.	The study involved healthy infants aged 3-6 months. Thirty participants completed the study. The intervention was the addition of an emollient to a skincare regimen compared with the use of a cleanser alone. Evaluated for 5 weeks of study.	The results of the study showed that the use of an emollient in addition to a cleanser had a positive effect on the infant skin microbiome, leading to an increase in richness and diversity.
11.	Gadermann et al. (German) [24]	Probiotic baths for atopic dermatitis (2021)	This study investigated the efficacy of a probiotic bath additive on clinical symptoms and skin microbiome of patients with atopic dermatitis.	Twenty-two patients aged between 5 and 71 years applied a 10-min partial bath with 4.5 × 10^9^ or 9 × 10^9^ CFU of viable lactic acid bacteria per liter daily over a period of 14 days.	The results of the study showed a significant improvement in the clinical symptoms of atopic dermatitis. The SCORAD decreased significantly throughout the study. The mean SCORAD reduced from 63.04 on day 0 to 47.09 on day 7 and further to 35.26 on day 14.
12.	Kwon et al. (Korea) [25]	Changes in lesional and non-lesional skin microbiome during treatment of atopic dermatitis (2019)	This study aimed to evaluate changes in the skin surface microbiome in atopic dermatitis. The effect of narrowband ultraviolet B phototherapy was also studied to determine the influence of exposure to ultraviolet.	Subjects: 18 patients with atopic dermatitis. The intervention involved two types of treatments: one group was treated with NB-UVB+TCS, while the other group was treated with TCS only.	The results of the study showed that both treatment groups, those treated with NB-UVB+TCS and those treated with TCS, showed significant improvement in their TISS and EASI scores during the treatment period. NB-UVB phototherapy may have potential benefits in reducing eczema recurrence and increasing non-lesional microbial diversity.
13.	Beck et al. (New York) [26]	Tralokinumab treatment improves the skin microbiota by increasing the microbial diversity in adults with moderate-to-severe atopic dermatitis: Analysis of microbial diversity in ECZTRA 1, a randomized controlled trial (2023)	To explore the impact of tralokinumab on the skin microbiota by analyzing the lesional skin of adult individuals diagnosed with moderate-to-severe atopic dermatitis who participated in the phase 3 ECZTRA 1 trial (NCT03131648).	Total sample: 802 participants who were randomly selected with an average age of 38.8 years, *S. aureus* abundance data was taken from 780 participants, and microbiome abundance and profiling were taken from 84 participants (59 on tralokinumab and 25 on placebo). The intervention in this study was the treatment with tralokinumab.	Treatment with tralokinumab led to a greater reduction in *S. aureus *absolute abundance in lesional skin relative to placebo at week 16. It is also suggested that neutralizing IL-13 may enable a more effective innate immune response, potentially reducing *S. aureus* adhesion to corneocytes.
14.	Nakatsuji et al. (USA) [27]	Development of a human skin commensal microbe for bacteriotherapy of atopic dermatitis and use in phase 1 randomized clinical trial (2021)	To evaluate the safety and efficacy of a targeted therapy in atopic dermatitis that seeks to specifically work against *S. aureus*, but not act broadly against CoNS.	Adult patients (n = 54) with moderate-to-severe atopic dermatitis who were *S. aureus* culture positive at screening. The intervention involved the application of a targeted therapy that works specifically against *S. aureus* by reintroducing a protective strain of CoNS.	Eczema severity was not significantly different when evaluated in all participants treated with ShA9. Still, a significant decrease in *S. aureus *and increased ShA9 DNA were seen and met secondary endpoints. Some *S. aureus* strains on participants were not directly killed by ShA9. Still, expression of mRNA for PSMα was inhibited in all strains. These observations demonstrate the safety and potential benefits of bacteriotherapy for atopic dermatitis.

## Review

Classification of skin microbiota

The human skin harbors various microorganisms, including bacteria, fungi, and viruses, collectively called the skin microbiota. Like the microorganisms in our gastrointestinal tract, the bacteria found on our skin have crucial functions in defending against harmful pathogens, supporting the immune system, and facilitating the decomposition of natural substances [28]. Human skin sites can be classified based on physiological attributes, specifically their sebaceous (oily), moist, or dry nature [29].

**Figure 4:**
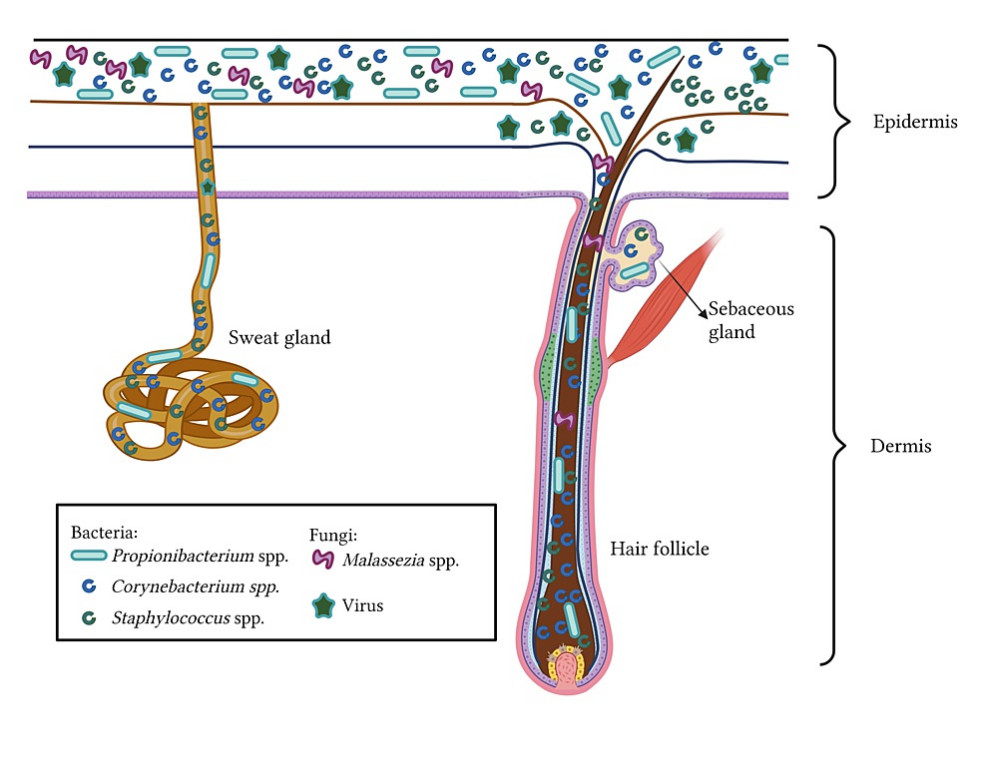
The sweat glands. It contains antimicrobial molecules, including free fatty acids and antimicrobial peptides, which effectively impede the colonization of microorganisms, which inhibit microbial colonization that causes the skin to have various moisture. This figure was created by NWM, one of the authors of this article with Biorender.com.

The skin comprises two layers: the epidermis and the dermis (Figure 4). The epidermis, which is the outermost layer, is composed of many layers of specialized keratinocytes. In conjunction with its stratified composition, the skin encompasses diverse microenvironments, contributing to its varying wetness levels. The sebaceous glands are responsible for sebum secretion, a substance rich in lipids. Sebum is a hydrophobic coating that lubricates and offers an antimicrobial protective layer to hair and skin [30].

Examining the microbial makeup at various anatomical sites is important in understanding the origins of prevalent dermatological conditions. It is believed that over one billion bacteria, comprising over 500 distinct species, inhabit each square centimeter of human skin. The composition of normal skin microbiota typically encompasses a minimum of 19 phyla (Tabel 2) [31].

**Table 2:** Normal human skin microbiome.

Skin type	Normal microbiome (most abundant bacterial group)
Sebaceus skin	Propionibacteria spp., Corynebacteria spp., other Actinobacteriales spp., and Staphylococci spp.
Moist skin	Corynebacteria spp., Staphylococci spp. Β- Proteobacteria, and γ-Proteobacteria
Dry skin	Β-Proteobacteria, Corynebacteria spp., Flavobacteriales

The microbiota exhibits variations across different anatomical sites, and the microbiome inside an individual can undergo temporal changes [32].

Humans' initial colonization of commensal microorganisms occurs during natural childbirth and the immediate postnatal period. Before birth, there is a notable surge in the population of Lactobacillus spp. within the vaginal region, which subsequently establishes the initial colonization of the microbiota throughout the process of natural childbirth. Following the cycle of birth, the neonate acquires supplementary microorganisms from healthcare professionals, parents, extended family members, and other individuals who interact with the infant. The procedure above initiates the development of a microbiome that will undergo ongoing changes throughout an individual's lifespan as novel microorganisms establish themselves and others are eradicated from the body [33].

Composition of the skin microbiome

The skin microbiome exhibits significant diversity regarding both organism abundance and metabolic function. Furthermore, the skin microbiota has temporal fluctuations as individuals age [34]. The microbiome is subject to various environmental influences, including but not limited to dietary patterns, antibiotic administration, and the presence of obesity [35].

The microbiota can be broadly categorized into two main groups: residents and transients. Variations in the makeup of the skin microbiota can be observed concerning age and gender [36]. The microbiota in the skin refers to bacteria that exhibit a persistent nature, remaining localized in some areas of the body and showing a temporal relationship with the individual's age. Research findings indicate significant variations in the occurrence of bacteria such as Streptococcus, Haemophilus, Neisseria, and other related species when comparing various individuals [37]. Transient microbiota refers to bacteria derived from the surrounding environment that do not establish a permanent presence [36]. During adolescence, the composition of the skin microbiota is primarily characterized by the prevalence of lipophilic bacteria [38].

The skin microbiome exhibits significant diversity regarding both organism abundance and metabolic function. Furthermore, the skin microbiota has temporal fluctuations as individuals age [34]. The microbiome is subject to various environmental influences, including but not limited to dietary patterns, antibiotic administration, and the presence of obesity [35].

Microbiome and skin barrier interactions

The skin microbiota and the skin barrier have a symbiotic relationship wherein they mutually influence each other through physical, chemical, and immunological mechanisms. The microbiome directly interacts with pathogenic bacteria encountered on the skin's surface. The physical barrier is the initial defense against external pathogens and other threats. The proliferation and differentiation of keratinocytes during the wound-healing process facilitate the reconstruction of the protective barrier against microbes [39]. An additional function of the microbiome involves the secretion of the constituents responsible for forming the lipid framework [40].

The skin's chemical barrier is established through the epidermis and the microbiome's secretion of various lipids and acids [41,42]. Some lipids, or free fatty acids, can inhibit harmful germs directly. In general, the structure and operation of the microbiome work in conjunction with the chemical barriers established by lipids and fatty acids present in the skin [43].

The microbiome elicits various innate immune responses and establishes symbiotic associations with the skin in cases where the skin barrier is compromised [44]. The distinct attributes of immune reactions can be initiated by microbial metabolic and inflammatory factors [45]. The correlation between adaptive immunity and microorganisms holds significant importance in developmental processes [46].

In conjunction with microbe-host interactions, microbe-microbe interactions are a protective mechanism against the invasion, colonization, and infection caused by invading, pathogenic, or opportunistic microorganisms. The interactions between microorganisms ensure their survival and enable them to maintain their specific ecological niche and access essential resources. The antimicrobial competition between these microorganisms significantly contributes to preserving homeostasis within the skin microbiome [47].

Accumulating data have proven the existence of reciprocal relationships between several barrier sites, such as the skin, intestines, lungs, and brain. The immune system in both the digestive tract and integumentary organs continues to work to maintain homeostasis, a state of internal balance. This phenomenon is commonly called the intestinal skin axis [48]. Skin problems can impact the composition and function of the gut microbiome. For example, the gut microbiome can be influenced by exposure to narrow-band ultraviolet light (NB-UVB) [49]. In addition, it should be noted that food allergies may arise due to exposure to epidermal proteins found in household dust. This exposure may ultimately trigger the growth of mast cells in the intestine via an immunoglobulin E-mediated mechanism [50].

In research conducted by O'Neill et al., it was observed that gut microbiota and their metabolites are potentially absorbed into the systemic circulation when there is a disruption in the gut barrier of individuals with psoriasis. These absorbed substances can then reach the skin and influence the skin's barrier function [51].

Human clinical trials conducted by Ogawa et al. and Chen et al. demonstrated that administration of Lactobacillus via oral supplementation resulted in a significant reduction in transepidermal water loss (TEWL), which serves as a reliable measure of skin barrier function [52]. The gut microbiome plays a vital role in restoring the integrity of the compromised skin barrier by being involved in innate and adaptive immune mechanisms [53].

Microbiome and atopic dermatitis

Skin Microbiome in AD

**Figure 5:**
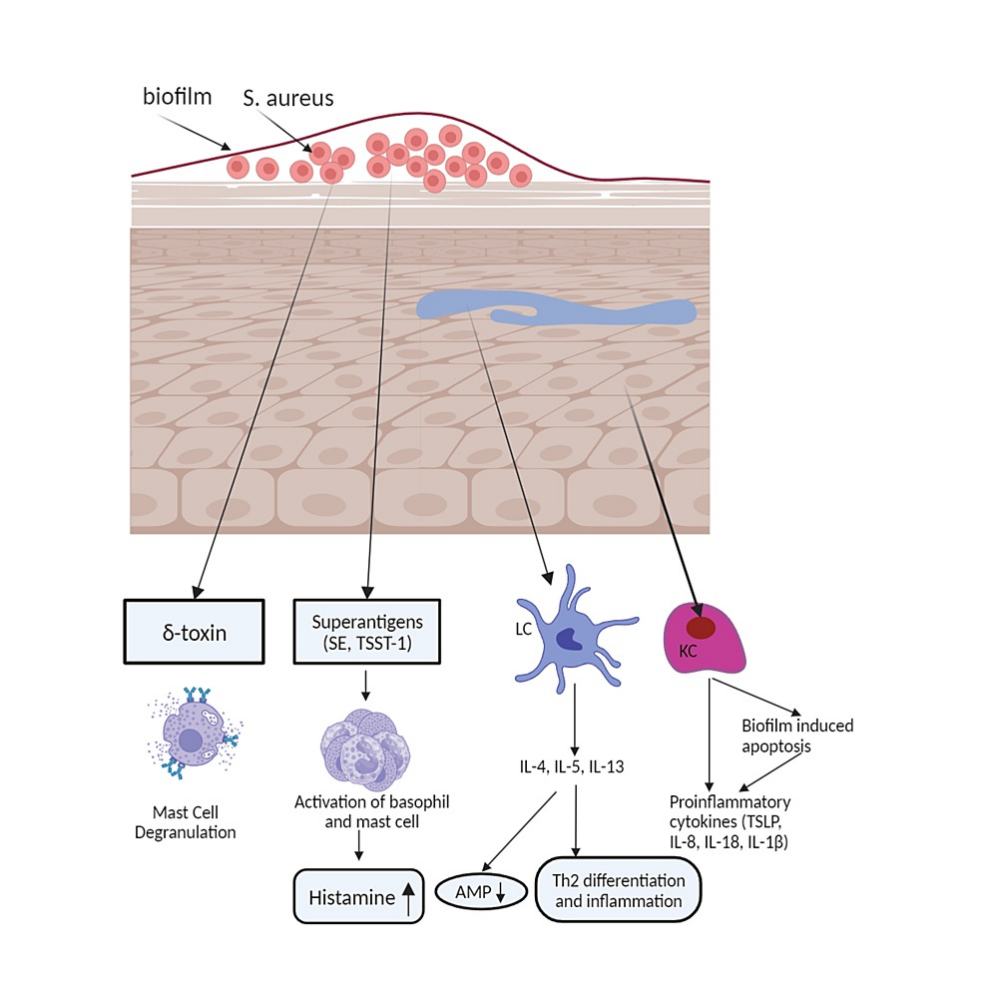
Atopic dermatitis can be caused by Staphylococcus aureus. *Staphylococcus aureus* produces superantigens like staphylococcal enterotoxins (SEs) and toxic shock syndrome toxin 1 (TSST-1), activating basophils and mast cells and releasing histamine. Langerhans cell (LC) is activated, releasing interleukin-4 (IL-4), interleukin-5 (IL-5), and interleukin-13 (IL-13), causing antimicrobial peptides (AMP) reduction, T-helper2 differentiation, and inflammation. Keratinocyte (KC) is stimulated, releasing proinflammatory cytokines like thymic stromal lymphopoietin (TSLP), interleukin-8 (IL-8), interleukin (IL-18), and interleukin-1 beta (IL-1β). Biofilm induces keratinocyte (KC) apoptosis, releasing inflammatory cytokines. *S. aureus* can also release delta toxin (δ-toxin), causing mast cell degranulation. This figure was created by AA, one of the authors of this article with Biorender.com.

*Staphylococcus epidermidis*, a Gram-positive bacterium, is the prevailing species found on the surface of healthy skin. It can impede the proliferation of *S. aureus*, another Gram-positive Staphylococcus species [54]. An imbalance in the skin microbiome has been identified as a potential risk factor for the development of AD [55]. The prevalence of *S. aureus* and *S. epidermidis* tends to rise in AD lesions [56]. *S. aureus* can directly hinder the function of the epidermal barrier by inducing the synthesis of endogenous serine proteases in keratinocytes. The process above leads to a decrease in FLG and other proteins found in the epidermis, thereby resulting in compromised lipid lamellae functionality, as shown in Figure 5 [57]. The occurrence of recurrent methicillin-resistant S. aureus (MRSA) infections can be observed in patients diagnosed with AD [58].

Alterations in the skin microbiome are particularly significant throughout the early stages of development, characterized by the relative immaturity of both the skin barrier function and the immune system [59].

Changes in Microbiota Colonization in AD

Dysbiosis refers to an alteration or disruption in the composition of the skin microbiota, leading to the development of pathogenic diseases [31, 32]. The understanding of the mechanism behind skin microbiota dysbiosis and its contribution to the development of AD is currently under active investigation.

**Figure 6:**
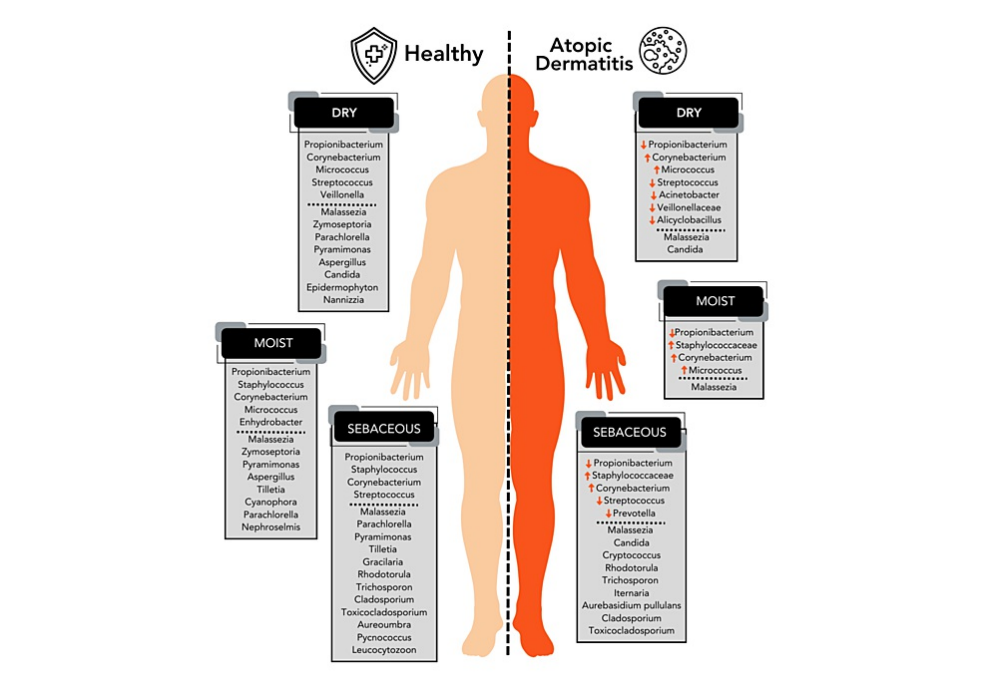
Comparison of microbiota composition between healthy skin and skin affected by atopic dermatitis. This figure was created by NA, one of the authors of this article on Biorender.com.

One of the proposed ideas about AD is the presence of dysbiosis in the microbiota residing on the skin surface, which is believed to be a consequence of barrier impairment resulting from genetic mutations in the filaggrin (FLG) gene [37]. Furthermore, it is worth noting that AD is associated with a reduction in ceramide levels. As long-chain free fatty acids, ceramides preserve moisture and serve as the primary water-retaining molecule inside the stratum corneum. A decrease in ceramide levels within the stratum corneum can lead to compromised skin permeability and an elevation in transepidermal water loss, resulting in the manifestation of dry skin. The occurrence of gene mutations and decreased ceramide levels in AD leads to skin barrier disruption [37]. The dysregulation of the skin's protective barrier leads to an imbalance in the composition of the microbiota, mainly resulting in an elevated presence and infiltration of *S. aureus* into the underlying skin layers. AD exacerbations are associated with a high population of *S. aureus* on the skin of affected individuals. Failure to treat exacerbations will result in an escalation of the *S. aureus* population, a decrease in microbial diversity, and the initiation of subsequent exacerbations [10]. Figure 6 illustrates the observed augmentation in microbial diversity inside healthy AD skin and AD lesions. During the period of exacerbation, it is frequently observed that there is an elevated level of colonization by *S. aureus* and *S. epidermidis* [13].

Microbiome in the treatment of atopic dermatitis

Literature Review Therapies Targeting Skin Microbiome

Based on the available microbiome research data, it is imperative to examine therapeutic considerations and strategies for preventing the recurrence of AD in the future.

A substantial body of information demonstrates a correlation between diminished epidermal barrier functionality and compromised skin microbiome composition. The utilization of antibiotics and antiseptics has been seen to reduce the presence of *S. aureus* on the skin, although it does not exhibit any notable enhancements to the overall microbiome. In contrast, the utilization of topical therapies containing corticosteroids, calcineurin inhibitors, moisturizers, and emollients has the potential to reinstate the integrity of the skin barrier and regulate the composition of the skin microbiota. Microbiota transplantation from healthy individuals could serve as a viable approach to repairing the skin microbiome in individuals with AD. There is a notable distinction in the Gram-negative microbiome of the skin between individuals with AD and those considered healthy controls.

In the study conducted by Auria et al. (Italy), fermented rice flour obtained from heat-killed Lactobacillus paracasei CBA L74 or placebo was administered once daily for 12 weeks to infants aged 6-36 months with moderate to severe atopic dermatitis. This therapy is given in addition to topical corticosteroid treatment. The evaluation included the primary outcome in the SCORAD index from baseline to 12 weeks. Secondary outcomes were gut microbiota composition, evaluated through analysis of stool samples and serum cytokines at the beginning and end of the intervention period in both groups, and steroid use during the treatment period and one month after treatment cessation. The V3-V4 region of the 16S ribosomal RNA gene was sequenced to evaluate changes in the gut microbiota. The research findings are as follows: no statistically significant variations were seen in cytokine levels, the makeup of the gut microbiota, or the relative abundance of bacterial genera across the groups. The use of topical steroids, measured as fingertips units, decreased from 4 to 16 weeks in both groups; the reduction was significantly higher in the experimental group than in the placebo group (p-value from the Wilcoxon rank sum test = 0.031). However, the results did indicate a potential steroid-sparing effect, which warrants additional investigation to determine its true worth [14].

Dupilumab is a monoclonal antibody that specifically targets the interleukin-4 receptor alpha, a key component involved in the pathogenesis of AD. This therapeutic agent has demonstrated efficacy in alleviating the clinical manifestations and subjective experiences associated with moderate-to-severe AD. The findings of the study by Callewaert et al. (USA) revealed an increase in microbial diversity and a decrease in the abundance of *S. aureus* during the administration of dupilumab treatment. Significant alterations were observed in both nonlesional and lesional skin. The quantity of *S. aureus* was shown to decrease with the administration of dupilumab, which was honored to be associated with improvements in the clinical manifestations of AD and biomarkers indicative of type 2 immune responses. The findings of this study indicate that the clinical amelioration of AD, achieved by inhibiting the interleukin-4 receptor a and the resultant mitigation of type 2 inflammation, is associated with heightened microbial diversity and a diminished prevalence of *S. aureus* [15].

The study conducted by Carucci et al. examined the potential of probiotics as a therapeutic intervention for AD, referred to as probiotics for Alzheimer's disease therapy (ProPAD). The objective of the ProPAD trial was to examine the therapeutic efficacy of the probiotic Lacticaseibacillus rhamnosus GG (LGG) in pediatric patients diagnosed with AD. A total of 100 patients diagnosed with AD and aged between 6 and 36 months were randomly assigned to receive either a placebo (group A) or LGG at a dosage of 1 × 10^10 colony-forming units (CFU) per day (group B) for 12 weeks. The findings revealed that the proportion of subjects who achieved the minimum clinically relevant difference (MCID) was significantly more significant in group B compared to group A (p < 0.05). The Infant Dermatitis Quality of Life questionnaire (IDQOL) showed significant improvement in group B (p < 0.05). The study findings revealed that group B patients exhibited a favorable alteration in both the gut and skin microbiota. At the same time, no such modulation was detected in other patient groups. The utilization of the probiotic LGG has the potential to serve as a beneficial supplementary treatment in the management of pediatric AD. The observed positive impacts on disease severity and quality of life were shown to be accompanied by a favorable alteration of the gut and skin microbiota [16].

In a study conducted by Smits et al., the efficacy of coal tar treatment for AD was examined, leading to a reduction in Staphylococcus levels and an increase in Propionibacterium populations. The therapeutic mechanism of coal tar, which was previously unknown, has been discovered to include the activation of the aryl hydrocarbon receptor, leading to the production of antimicrobial peptides generated from keratinocytes. Creating an antimicrobial environment that is less susceptible to infection and inflammation can provide advantageous outcomes [17].

Staphefekt, the modified bacteriophage endolysin, demonstrates a targeted ability to eliminate *S. aureus* bacteria while preserving the presence of other commensal microorganisms on the skin. The phenomenon of bacterial resistance to endolysins has yet to be documented in scientific literature, and it is not anticipated based on current knowledge [18]. This absence of resistance allows Totte et al. to investigate the potential of endolysins as a sustained treatment for Staphylococcus infections in individuals with non-infected AD. The trial's findings yield information regarding the impact of extended anti-staphylococcal treatment using Staphefekt on using corticosteroids, clinical symptoms, and quality of life in individuals diagnosed with moderate to severe AD. Further information regarding the growth features of the skin microbiome, namely *S. aureus*, will provide valuable insights into the microbiome's involvement as a contributing element in the pathophysiology of AD [18].

The study results from Zelenkova et al. showed that the regular application of the emollient 'plus' led to a considerable decrease in the usage of corticosteroids compared to the control group. Emollient Plus is an emollient preparation in the form of a light balm containing vitamin E, tocopherol, and glycerin and enriched with Aqua posae filiformis and microresil under the trademark LIPIKAR BAUME LIGHT AP+M, produced by La Roche-Posay Laboratoire Dermatologique. The result implies that the emollient 'plus' has the potential to serve as a viable intervention for the management of dermatitis and to decrease the need for corticosteroid usage [19].

Weis et al. (Denmark) [20] investigated the effectiveness of topical niclosamide (ATx201) in reducing *S. aureus* colonization and increasing skin microbiome diversity in atopic dermatitis patients. The study population consisted of 40 atopic dermatitis patients, consisting of male and female subjects aged between 18 and 70 years. The intervention used ATx201 2% ointment, a topical formulation of the drug niclosamide, traditionally used to treat tapeworm infections. The ointment is applied to certain treatment areas on the patient's skin. ATx201 is a potent agent against MRSA and other *S. aureus* strains, including those resistant to commonly used topical treatments. The study findings demonstrated that ATx201 showed significant efficacy against MRSA and various strains of *S. aureus*, including those that displayed resistance to routinely employed topical therapies. The compound showed bacteriostatic killing kinetics at lower concentrations; however, at a greater concentration, it effectively eradicated the bacterial population.

Youssef et al. administered treatment with NB-UVB or glycerol at 85% to individuals who had mild to moderate AD for one month and then observed them for an additional month. Samples were collected from the skin and nasal passages to cultivate Staphylococci on mannitol-salt agar. This cultivation aimed to quantify the number of CFU. Both groups had microbiological alterations that were not statistically significant while demonstrating clinical improvement that was statistically significant following treatment. Concentrated glycerol at an 85% concentration presents itself as a cost-effective, efficient, and easily obtainable substitute for phototherapy for individuals with mild to moderate AD who are unable to avail themselves of the necessary medical facility. The reduction of staphylococcal skin load is a factor that is implicated, albeit to a limited extent [21].

The study by Zeng et al. (China) investigates the comparative effects of topical ozone hydrotherapy followed by ozonated oil therapy concerning tap water and basal oil on AD skin lesions. Following a three-day ozone therapy intervention, patients exhibited a notable reduction in SCORAD scores and a decrease in the presence of inflammatory cells infiltrating AD lesions. The non-lesional areas showed greater micro-ecological diversity than the lesional areas (p < 0.05). There was a positive correlation between the proportion of *S. aureus* in AD lesions and the severity of AD (r = 0.564, p = 0.010), which showed a decrease following ozone therapy (p = 0.07). The application of ozone therapy demonstrated a notable augmentation in microbiological diversity, accompanied by a statistically significant rise in the fraction of Acinetobacter (p < 0.05). The efficacy of topical ozone therapy in the treatment of AD is substantial. The alteration of the proportionate ratio between Staphylococcus and Acinetobacter can potentially restore the microbial variety observed in lesions associated with AD [22].

Capone et al. (USA) investigated the effects of emollient use on the microbiome and metabolome of developing infant skin [23]. The study involved healthy babies aged three to six months. Thirty participants completed the study. The intervention is the addition of emollients to the skin care regimen compared to using cleanser alone. They were evaluated for five weeks. The study found that the wash-plus lotion group had significantly greater microbial richness compared to the wash-only group. This suggests that the inclusion of lotion in a healthcare routine can benefit the microbiome of infant skin. The initial stage in achieving enhanced diversity linked to optimal skin health is an elevation in overall richness. In the group that received both wash and lotion, there was a decrease in protein breakdown compared to the group that only received wash. Additionally, the levels of important components of the skin barrier function, such as ceramides, rose compared to the initial baseline levels [23].

Gadermann et al. (Germany) conducted research using probiotic bath treatments for atopic dermatitis [24]. This study investigated the efficacy of probiotic bath additives on the clinical symptoms and skin microbiome of patients with atopic dermatitis. The present study examined the effects of topically applying probiotic dietary supplements containing nine strains of bacteria, either as a bath or a partial bath, on the modulation of the dermal and epidermal immune systems. Twenty-two patients aged between 5 and 71 years took 10-minute partial baths with 4.5 × 10^9^ or 9 × 10^9^ CFU of active lactic acid bacteria per liter daily for 14 days. The objective was to assess the potential positive impact on barrier function and the inhibition of pathogenic species through species antagonism. Using a probiotic bath topically suggests a potentially effective therapeutic approach for AD, offering relief from the current imbalance of microbial communities. The results showed a significant improvement in the clinical symptoms of atopic dermatitis [24].

In their study, Kwon et al. examine the alterations in the microbiota of both lesional and non-lesional skin throughout AD treatment [25]. NB-UVB therapy has been shown to have advantages in mitigating eczema exacerbations and enhancing microbial diversity in non-lesional areas. Ultraviolet radiation exposure has been found to stimulate the synthesis of the cathelicidin peptide LL-37 in the skin affected by AD. The findings of this study indicate that individuals who received NBUB treatment saw a reduced number of relapses. Other researchers also reported similar results. According to a study conducted in the United Kingdom, children with AD who received NB-UVB phototherapy had superior clinical outcomes compared to the control group, with these benefits persisting for six months post-phototherapy. The occurrence of this enduring therapeutic impact is infrequently observed in alternative treatment approaches. The skin in AD lesions has a significantly greater abundance of *S. aureus* and a reduced level of microbial diversity. The amelioration of eczema is associated with a significant decrease in the presence of *S. aureus* within skin lesions. NB-UVB treatment has been observed to effectively mitigate eczema flare-ups while concurrently enhancing microbial diversity in non-lesional areas [25].

Research conducted by Beck et al. (New York) is administering tralokinumab ECZTRA 1 (NCT03131648) treatment to improve skin microbiota by increasing microbial diversity in adults with moderate to severe atopic dermatitis [26]. Treatment with tralokinumab resulted in a more significant reduction in the absolute abundance of *S. aureus* in skin lesions compared with placebo at week 16. It has also been suggested that neutralizing interleukin-13 (IL-13) may allow a more effective innate immune response, thereby potentially reducing Staphylococcus aureus adhesions to corneocytes [26].

Nakatsuji et al. (USA) developed human skin commensal microbes for bacteriotherapy of atopic dermatitis and used them in a phase 1 randomized clinical trial [27]. This current study aimed to examine the safety and elucidate the mechanisms of action of *S. hominis* A9 (ShA9), a bacterium derived from the skin of healthy individuals, in its potential application as a topical treatment for AD. The administration of ShA9 exhibited inhibitory effects on the expression of psmα, a toxin produced by *S. aureus* known to induce inflammatory responses. The primary objective of a phase 1 clinical trial (NCT03151148) was to assess the safety of topical application of ShA9 compared to a control vehicle on the forearm skin of 54 individuals diagnosed with AD who tested positive for *S. aureus*. The experiment followed a double-blinded, randomized design and lasted for one week. The results demonstrated that ShA9 was well-tolerated, meeting the primary endpoint of safety. Furthermore, participants who received ShA9 experienced a lower incidence of adverse events associated with AD. ShA9 did not effectively eradicate certain strains of *S. aureus* in the study participants. However, it was shown that messenger RNA (mRNA) expression for psmα was universally suppressed in all strains. The post-hoc examination of subjects with *S. aureus* directly eliminated by ShA9 indicated a potential improvement in the severity of local eczema. These observations indicate the safety and potential benefits of bacteriotherapy for atopic dermatitis. These observations demonstrate bacteriotherapy's safety and potential benefits for atopic dermatitis [27].

## Conclusions

The dysregulation of the skin microbiota contributes to the worsening of AD, leading to increased colonization of pathogenic bacteria, particularly *S. aureus*, which significantly impacts AD progression. Ongoing research is being conducted to further explore the involvement of bacteria in dermatology, with a particular focus on AD. Various therapies that target the microbiome of the skin to treat atopic dermatitis include fermented rice flour obtained from heat-killed *Lactobacillus paracasei* CBA L74, monoclonal antibody Dupilumab, probiotic *Lacticaseibacillus rhamnosus*, coal tar, Staphefekt, the modified bacteriophage endolysin, emollient 'plus', topical niclosamide, NB-UVB or glycerol at 85%, topical ozone hydrotherapy followed by ozonated oil, probiotic bath treatment, narrowband UVB therapy (NBUVB), tralokinumab ECZTRA 1 (NCT03131648), and application of *S. hominis* A9. The potential significance of the skin microbiota in the context of AD warrants attention for future treatment strategies targeting AD.
